# Cancer as a risk factor for distress and its interactions with sociodemographic variables in the context of the first wave of the COVID-19 pandemic in Germany

**DOI:** 10.1038/s41598-022-06016-x

**Published:** 2022-02-07

**Authors:** Mareike Ernst, Manfred E. Beutel, Elmar Brähler

**Affiliations:** 1 Department of Psychosomatic Medicine and Psychotherapy, University Medical Center of the Johannes-Gutenberg-University Mainz, Mainz, Germany; 2 Integrated Research and Treatment Center Adiposity Diseases, Behavioral Medicine Research Unit, Department of Psychosomatic Medicine and Psychotherapy, University of Leipzig Medical Center, Leipzig, Germany

**Keywords:** Psychology, Oncology, Risk factors, Signs and symptoms, Cancer, Psychiatric disorders, Public health, Epidemiology

## Abstract

The COVID-19 pandemic poses a psychological challenge, especially for individuals with chronic illnesses. The aim of this study was to investigate associations of cancer with distress, including its interplay with further risk and protective factors. We conducted a representative survey of the German population (*N* = 2503, including *N* = 144 with a cancer diagnosis) during the first wave of the pandemic. In multiple linear and logistic regression analyses, we tested associations of cancer with depression and anxiety symptoms and suicidal ideation. We also investigated moderating effects of age, gender, income, living situation, marital status, and loneliness. Individuals with cancer were more likely to report anxiety symptoms (*φ* = .061), suicidal ideation (*φ* = .050), and loneliness (*φ* = .044) than other participants. In regression analyses that controlled for sociodemographic differences, cancer was still associated with anxiety symptoms. We also observed interaction effects, indicating that this relation was especially strong in men with cancer and that cancer survivors with a low income were particularly likely to report anxiety symptoms. The findings demonstrate that cancer survivors are a vulnerable group and that factors of different life domains interact in shaping well-being in the population, necessitating comprehensive risk assessment and support offers during the pandemic and beyond.

## Introduction

In March 2020 the World Health Organisation declared the spread of SARS-CoV-2, the virus causing coronavirus disease 19 (COVID-19), a pandemic. Since then, many countries around the world have implemented unprecedented prevention measures. The closure of educational institutions and childcare facilities, cuts in the labour market, and far-reaching contact restrictions have led to an ongoing stagnation of public life. Against this background, potential mental health sequelae of the COVID-19 pandemic which could constitute a public health crisis in their own right have come into focus^1,2^.

Although first empirical evidence has shown little to moderate overall increases in psychological distress^3–5^, the pandemic has also aggravated existing disparities as risk factors for distress during the pandemic corresponded to those observed before its outbreak. For instance, internalizing problems such as depression and anxiety symptoms were more common among those with lower socioeconomic status, women, and individuals who reported being socially isolated^4,6–8^. Furthermore, social isolation and low household income were risk factors for loneliness in a large population study in the UK^9^.

A particular group at risk for unfavorable outcomes are individuals with pre-existing medical conditions^10,11^, including common diseases such as cancer. The National Cancer Institute estimates that almost 40% of men and women will receive a cancer diagnosis in their lifetime^12^. Cancer is now seen as a chronic disease many people can live with for years or even decades due to better diagnostic and treatment options^13^, but the relations of cancer with mental health are complex: In many studies, cancer patients and long-term survivors^14^ were more likely than the general population to suffer from anxiety and depression symptoms^15–17^. However, researchers have also warned that especially questionnaire instruments that include items assessing somatic complaints (such as the PHQ-9) could overestimate rates of mental distress in cancer patients/survivors due to their overlap with symptoms of the disease and/or side effects and late effects of its treatment, e.g., tiredness and low energy^18,19^.

Furthermore, there are considerable differences *within* cancer patient/survivor samples as the implications of cancer for mental health are also shaped by other risk factors or resources a person has, respectively, such as their financial means, social support, living situation, etc.^20–22^. Along these lines, increased psychological morbidity was no universal finding of large-scale, population-representative investigations from Germany and the United States^22–24^. Researchers have even reported positive psychological outcomes, e.g., under the umbrella term “posttraumatic growth”^25,26^.

However, there is no dispute that the COVID-19 pandemic implicates a host of new, specific stressors for cancer patients and survivors such as disruptions and/or delays of cancer treatment, bans on companions or visitors, and generally worse access to health-care services^27,28^. Moreover, research findings indicate that they are more likely than individuals without pre-existing medical conditions to experience a severe course of illness and/or die from COVID-19 if infected^29,30^. This current threat situation and the increased salience of severe disease and death in the public discourse^31^ challenge cancer patients’ and survivors’ mental health.

So far, the bulk of mental health research conducted in the context of the COVID-19 pandemic has focused on the general population and health-care workers^4,32^. In comparison, information about cancer survivors’ well-being is scarce. A large US-based survey, which included 854 individuals with a self-reported cancer diagnosis, found that cancer survivors were more likely to report anxiety and depression symptoms than the rest of the population^33^. By contrast, in a study conducted at a German university hospital, cancer patients did not report higher levels of distress or anxiety (both COVID-19-related and -unrelated) than matched, healthy controls^34^. However, their mental health status deteriorated over a 2-week period during the start of the first wave of the pandemic^35^. These changes were statistically predicted by higher COVID-19-related fear and lower trust in the government. In a sample of 240 Austrian cancer patients surveyed in Spring 2020, female gender was another risk factor for greater distress^36^. While a longitudinal investigation of 518 cancer survivors in the UK statistically controlled for gender (as a potential confounding factor), it highlighted the detrimental effects of experiencing loneliness during the pandemic as it significantly increased the risk of depression symptoms^37^. Feelings of social isolation were especially pronounced in older cancer patients and those with lower educational attainment in an Italian survey^38^.

### The present study

The first aim of the present work was to compare cancer survivors’ mental health and feelings of loneliness with the general population using a representative community sample. We hypothesized that more cancer survivors would report clinically relevant symptom burden and loneliness than the general population.

Secondly, we aimed to ascertain the statistical relevance of a cancer diagnosis for anxiety and depression symptoms and suicidal ideation and to examine its interplay with other risk factors such as sociodemographic characteristics. As individual (mental) health is shaped by the intersection and simultaneity of different biological, social, and psychological risk and protective factors, we aimed to expand previous research by also testing moderating effects of e.g., gender, age, and income, with respect to the association of a cancer diagnosis and mental health outcomes. This approach also builds on the cited previous research that has highlighted the ways in which the pandemic affects people differently according to these factors.

We hypothesized that cancer survivors’ psychological vulnerability increased if they were women, lived alone, had a low income, were unmarried, and had a low level of education.

## Methods

### Survey method

Data was collected between April and June 2020 by the institute USUMA (Unabhängiger Service für Umfragen, Methoden und Analysen; German for: Independent service for surveys, methods and analyses). This period captured the first wave of the COVID-19 pandemic in Germany. Public health measures in place during this time included the closing of school and universities, travel restrictions, the closing of non-essential businesses, and bans of gatherings of more than two people. With respect to the medical domain, outpatient and inpatient diagnostic procedures and treatments were delayed or postponed (by the hospitals burdened with COVID cases and/or by patients afraid of contracting the virus) (e.g.,^39–41^).

The survey was approved by the institutional ethics review board of the University of Leipzig (number 043/20‐ek). All methods were carried out in accordance with the relevant guidelines and regulations. All participants were informed of the study procedures, data collection and anonymization of personal data before providing informed consent. Informed consent was obtained from all participants. If participants were younger than 16 years, consent was also obtained from a parent/legal guardian. Participating households and individuals were chosen using random route procedures and Kish selection^42^. In-person-interviews were carried out observing hygiene regulations (face masks and physical distance). Sociodemographic information was collected in the form of an interview. After this, participants completed the rest of the questionnaire independently in writing. The interviewer remained present to answer questions of comprehension.

Inclusion criteria were an age of 14 years or older and sufficient German language skills. Participants were individually interviewed at their home by a trained interviewer and completed several questionnaires. In total, 2503 individuals were questioned by trained interviewers (representing 46.5% of the 5418 households addressed). According to information obtained from the Federal Statistical Office, this analysis sample was representative for the German residential population.

### Measures

Sociodemographic information comprised participants’ birth date, gender, marital status, living situation, and income. The equivalized income was calculated according to the OECD guideline (Household income/√(people in household)). Monthly income was then categorized as follows: 1 =  < 1250€, 2 = 1250–2500€, 3 =  > 2500€^43^.

Anxiety symptoms were assessed using the GAD-2 (capturing nervousness and worrying)^44,45^. Depression symptoms were assessed using PHQ-2 (capturing depressed mood and anhedonia)^46,47^. The GAD-2 and PHQ-2 are widely used screening questionnaires using the same response format. Participants are asked to report how often they were bothered by the respective symptom over the course of the last two weeks. Response options are 0 = not at all, 1 = several days, 2 = more than half the days, 3 = nearly every day. The respective sum score thus ranges from 0 to 6. In order to report prevalence rates, we used the previously established cut-off of ≥ 3 for clinically relevant symptom burden. For the GAD-2, Plummer, et al.^45^ reported a pooled sensitivity of 0.76 and specificity of 0.81. For the PHQ-2, sensitivity was 0.76 and specificity was 0.87^48^. In the present sample, the GAD-2 (*ω* = 0.76) and PHQ-2. (*ω* = 0.72) showed good internal consistency.

We used a single item drawn from the depression module of the Patient Health Questionnaire PHQ-9^49^ to assess suicidal ideation (“Thoughts that you would be better off dead or of hurting yourself in some way”). The response format was the same as for the PHQ-2 and GAD-2 items. In line with previous research^50^, the item was recoded into a binary variable (0 = not bothered at all, 1 = combining several days, more than half the days, and nearly every day).

Loneliness was assessed with the UCLA 3-item loneliness scale^51^ capturing the subjective experience of lack of companionship, feeling left out, and isolated from others. Participants respond how often they feel this way on a Likert scale ranging from 0 = “never” to 4 = “very often”. The German version^52^ is typically scored from 0 to 12 and has previously shown to be measurement invariant with respect to gender and age^53^. In the present sample, its internal consistency was good (*ω* = 0.82). In line with previous research^54^ we used a cut-off of ≥ 5 to differentiate lonely and not lonely participants.

Cancer diagnoses were assessed via self-report (“Have you ever been diagnosed with cancer?”). Response options were “yes” and “no”. Participants were also asked to specify whether they had received a diagnosis during the last 12 months. In line with the definition of the National Cancer Institute^55^ (i.e., that the time of survival starts at time of diagnosis), we refer to all participants with cancer as cancer survivors within the following text.

### Analyses

Data were analyzed using univariate comparisons (independent *t* tests and χ^2^-tests, where appropriate). To assess the association of a cancer diagnosis with mental health outcomes, we also calculated two multiple linear regression analyses (of depression and anxiety symptoms) and one multiple logistic regression analysis (of suicidal ideation). Variables tested as statistical predictors comprised sociodemographic variables (age, gender, income, living situation, marital status) and loneliness. All metric predictors used in interaction terms (such as age) were mean-centered before. Age (in years), equivalized income, and loneliness were entered as continuous variables, marital status was coded 0 = not married, 1 = married, education was coded 0 = educational attainment below high school degree, 1 = high school degree and/or further education, living situation was coded 0 = living in a shared household, 1 = living alone. Including the interaction terms, each regression model contained 15 predictors. Therefore, for detecting small effect sizes (*f*^2^ = 0.02) with a statistical power level of 0.8, the minimum required sample sized was *N* = 950. For the logistic regression model of suicidal ideation, there were 235 cases available. Based on established rules of 10 events per variable (EPV), this was a sufficiently large sample size for a logistic regression model including 15 predictors^56^. Within the models, we checked the Variance Inflation Factor (VIF) for each independent variable. We observed no value above four, indicating no concerning level of multicollinearity^57^. In the following, regression coefficients and effect sizes are interpreted following Cohen^58^. P-values refer to two-tailed tests. All analyses were conducted using R version 4.0.3.

## Results

### Participants

In total, 2,503 individuals took part. Slightly more than half (*N* = 1329, 53.1%) were women. A cancer diagnosis was reported by 144 participants (5.8% of the sample). Among them were 89 women (61.8%). There were no statistically significant differences regarding mental health outcomes between those who had reported a cancer diagnosis during the last 12 months (*N* = 37) and those who had received a diagnosis longer ago (*N* = 107), which is why they were all grouped together for the purpose of the following analyses.

### Comparison of participants with and without cancer

**Figure 1:**
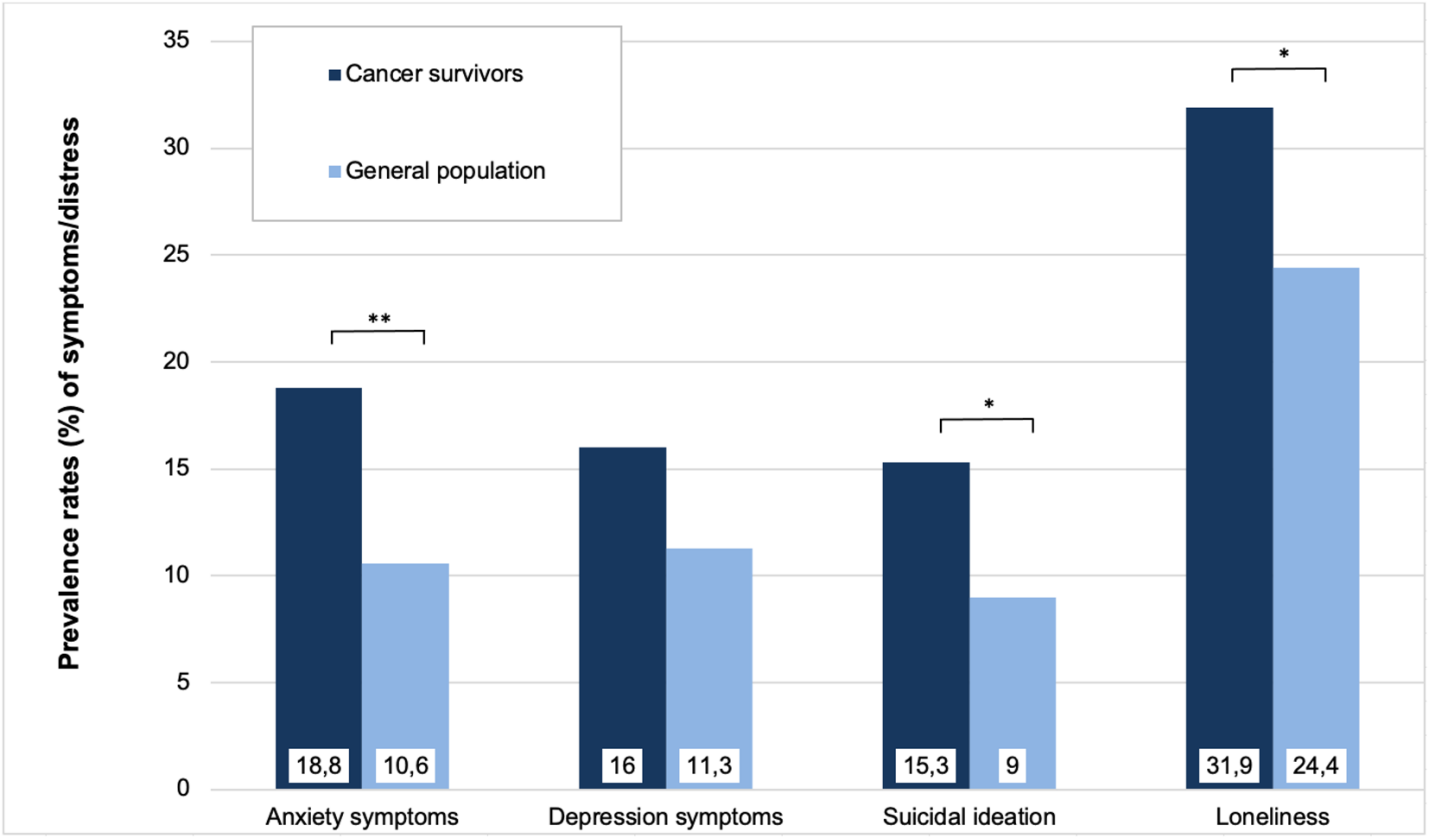
Prevalence rates of anxiety and depression symptoms, suicidal ideation, and loneliness in cancer survivors and the general population. Larger proportions of cancer survivors (dark blue) than of the general population (light blue) reported clinically relevant anxiety symptoms, suicidal ideation, and loneliness. With respect to depression symptoms, there was only a trend.

Univariate differences between cancer survivors and those without a cancer diagnosis are presented in Table 1. Cancer survivors were significantly older than the rest of the sample (*p* < 0.001, *d* = 0.8) and more of them were married (*p* = 0.004, *φ* = 0.06). Group comparisons with respect to the prevalence of psychological distress symptoms and loneliness are shown in Fig. 1. The proportions of individuals reporting anxiety symptoms (*p* = 0.006, *φ* = 0.061), suicidal ideation (*p* = 0.018, *φ* = 0.050), and loneliness (*p* = 0.035, *φ* = 0.044) were greater among cancer survivors than among the general population.

**Table 1:** Sample characteristics (stratified by self-reported cancer diagnosis).

	Participants without cancer (*N* = 2359)	Cancer survivors (*N* = 144)	*p*
*Sociodemographic information*			
Women, *N* (%)	1240 (52.6)	89 (61.8)	.096
Age, *M* (*SD*)	45.22 (17.61)	58.73 (15.40)	** < .001**
Married, *N* (%)	973 (41.2)	77 (53.5)	**.004**
High level of education^1^, *N* (%)	726 (30.8)	37 (25.7)	.23
Equivalized household income, *M* (*SD*)	1.56 (0.57)	1.57 (0.56)	.96
Living alone, *N* (%)	693 (29.4)	51 (35.4)	.13

Univariate comparisons are Chi-square or t-tests, where appropriate. Categorical data is shown as *N* and %, continuous variables as means and standard deviations. Significant values are in bold.

^1^German Abitur (12–13 years of school) or equivalent that allows for postsecondary education at universities.

### Associations of cancer and sociodemographic characteristics with mental health outcomes

We calculated two separate multiple linear regression analyses and one logistic regression analysis to test associations of cancer and sociodemographic characteristics with depression symptoms, anxiety symptoms, and suicidal ideation. Both linear models explained a highly significant proportion of the respective outcome’s variance (adjusted R^2^ = 0.31 and adjusted R^2^ = 0.35, respectively).

In the linear regression model of depression symptoms (Table 2), loneliness was the only statistically significant predictor (*β* = 0.59, *p* < 0.001). In this model, cancer had no relevant associations with the outcome.

**Table 2:** Multiple linear regression of depression symptoms (PHQ-2).

Predictors	Linear regression of depression symptoms		
	*B* (*SE*)	*β*	*p*
Gender	0.06 (0.04)	0.03	.16
Age	− 0.02 (0.03)	− 0.01	.48
Married	0.00 (0.06)	0.00	.99
High level of education	0.02 (0.05)	0.01	.75
Living alone	− 0.04 (0.06)	− 0.02	.52
Income	− 0.05 (0.02)	− 0.04	.062
Loneliness	0.72 (0.02)	0.59	** < .001**
Cancer	0.19 (0.42)	0.04	.65
Cancer × Gender	0.01 (0.20)	0.00	.96
Cancer × Age	− 0.03 (0.13)	− 0.01	.80
Cancer × Married	− 0.28 (0.25)	− 0.04	.27
Cancer × High level of education	0.20 (0.22)	0.02	.38
Cancer × Living alone	0.06 (0.27)	0.03	.83
Cancer × Income	− 0.07 (0.10)	0.01	.53
Cancer × Loneliness	− 0.07 (0.09)	− 0.02	.42

Adjusted R^2^ = 0.35. Significant values are in bold.

**Figure 2:**
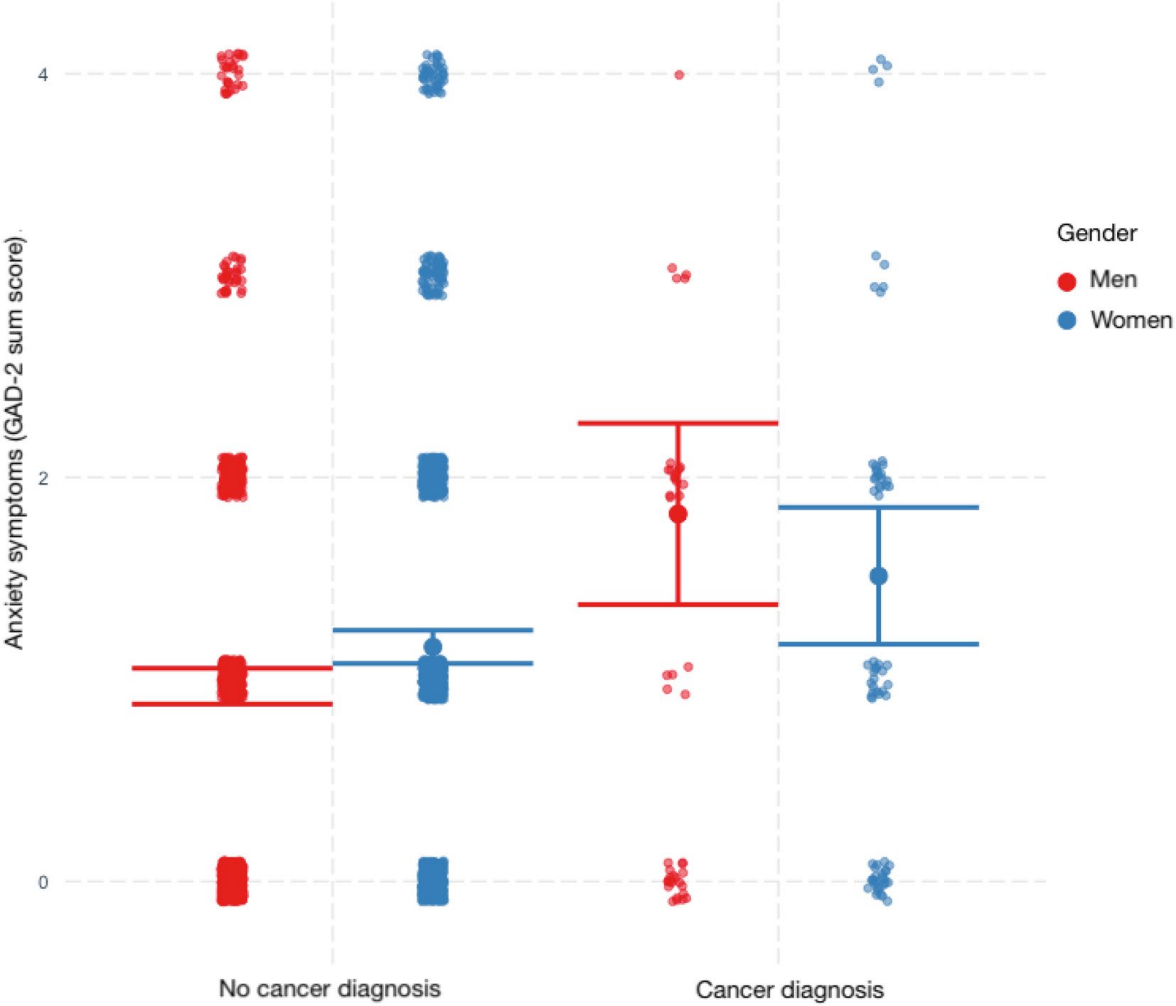
Levels of anxiety symptoms in men and women with and without cancer. The figure depicts the significant interaction effect of a cancer diagnosis and gender in the statistical prediction of anxiety symptoms within a multiple linear regression model controlling for other risk factors (including age, equivalized income, and living situation): Especially men with a cancer diagnosis were at risk to suffer from anxiety symptoms.

Table 3 shows the linear regression model of anxiety symptoms. Higher levels of anxiety symptoms were related to female gender (*β* = 0.07, *p* < 0.001), cancer (*β* = 0.24, *p* = 0.003), and loneliness (*β* = 0.53, *p* < 0.001). We also observed statistically significant interaction effects in the sense that cancer was a stronger predictor of anxiety symptoms in men compared to women and in individuals with a lower compared to a higher household income. The interaction of cancer and gender is visualized in Fig. 2.

**Table 3:** Multiple linear regression of anxiety symptoms (GAD-2).

Predictors	Linear regression of anxiety symptoms		
	*B* (*SE*)	*β*	*p*
Gender	0.20 (0.05)	0.07	** < .001**
Age	− 0.05 (0.03)	− 0.04	.060
Married	0.02 (0.06)	0.01	.75
High level of education	0.01 (0.05)	0.00	.90
Living alone	− 0.01 (0.07)	0.00	.89
Income	− 0.03 (0.03)	− 0.02	.26
Loneliness	0.69 (0.02)	0.53	** < .001**
Cancer	1.37 (0.46)	0.24	**.003**
Cancer × Gender	− 0.50 (0.22)	− 0.15	**.021**
Cancer × Age	− 0.04 (0.14)	− 0.01	.81
Cancer × Married	− 0.45 (0.28)	− 0.06	.11
Cancer × High level of education	0.24 (0.24)	0.02	.32
Cancer × Living alone	− 0.43 (0.30)	− 0.05	.15
Cancer × Income	− 0.25 (0.11)	− 0.02	**.030**
Cancer × Loneliness	0.13 (0.10)	0.03	.19

Adjusted R^2^ = 0.31. Significant values are in bold.

Lastly, within the multiple logistic regression model of suicidal ideation (Table 4), loneliness (*OR* = 1.35 [95% CI 1.28–1.43], *p* < 0.001) was related to a higher likelihood of reporting suicidal ideation. This was also the case for higher age (*OR* = 1.01 [95% CI 1.00–1.02], *p* = 0.02). However, we found no effects of cancer and no interactions of cancer with other variables within this model.

**Table 4:** Multiple logistic regression of suicidal ideation (PHQ-9 item).

Predictors	Logistic regression of suicidal ideation		
	*OR*	95% CI	*p*
Gender	1.04	0.76–1.43	.80
Age	1.01	1.00–1.02	**.02**
Married	0.71	0.48–1.07	.11
High level of education	0.98	0.69–1.39	.93
Living alone	0.78	0.51–1.19	.24
Income	0.86	0.64–1.18	.36
Loneliness	1.35	1.28–1.43	** < .001**
Cancer	5.87	4.12–19.54	.39
Cancer × Gender	0.30	0.08–1.13	.08
Cancer × Age	0.98	0.94–1.03	.42
Cancer × Married	0.48	0.10–2.92	.55
Cancer × High level of education	0.83	015–4.65	.83
Cancer × Living alone	1.34	0.24–7.50	.74
Cancer × Income	1.75	0.52–5.82	.36
Cancer × Loneliness	1.20	0.93–1.54	.16

Nagelkerke R^2^ = 0.25. OR = Odds Ratio. CI = Confidence Interval. Significant values are in bold.

## Discussion

The objective of the present study was to investigate potential differences in mental health outcomes of individuals with cancer and the rest of the general public during the first wave of the COVID-19 pandemic in Germany and to explore the interplay with other risk/protective factors which were previously found to play a relevant role in cancer patients’ and survivors’ mental health and in the emotional well-being of the general population in the context of the pandemic. To this aim, we used data from a representative community sample and calculated both unadjusted comparisons and multiple linear and logistic regression analyses that included several interaction terms.

In our study, individuals with cancer were more likely to report anxiety symptoms, loneliness, and suicidal ideation than other participants. The largest effect size was observed for the group comparison regarding anxiety symptoms (which comprised nervousness and worrying). This is in line with the results of a recent meta-analysis of studies carried out during the pandemic that corroborated higher levels of anxiety symptoms among cancer patients compared to control participants^59^. A previous representative study conducted in Germany that had assessed cancer patients and survivors as part of a large community cohort before the pandemic had found no overall differences between people affected by cancer and others with respect to current anxiety symptoms^22^. Similar to the present results, previous research conducted during an early phase of the pandemic had also reported higher rates of loneliness among cancer survivors^37^ compared to other members of the community in a large, population-based survey. However, in contrast to the already referenced study from the UK^37^ and a report from the USA^33^, we did not observe elevated rates of depression symptoms among individuals with cancer. Cancer patients’ and survivors’ levels of depression symptoms have already varied in research reports before the pandemic. As an example, comparatively large proportions of cancer patients fulfilling criteria of clinically relevant depression symptoms^60^ were contrasted by a longitudinal study within a prospective community cohort that controlled for other biopsychosocial risk factors and found that a cancer diagnosis did not predict depression symptoms (using the PHQ-9) over time^61^.

Besides anhedonia and depressed mood/hopelessness, the depression symptoms captured by the PHQ-2, the present study investigated suicidal ideation separately. It was more prevalent among cancer survivors. Unfortunately, we could not locate any other empirical research that assessed cancer survivors’ suicidal ideation during the COVID-19 pandemic and compared them to a population sample. This presents a research gap as studies conducted before the pandemic had described cancer survivors’ elevated risk for suicide outcomes including both suicidal thoughts and dangerous behaviors such as suicide attempts^62^. Given the relevance of social relationships, including feelings of loneliness, for suicidal crises^63,64^ and the fact that cancer survivors have reported to avoid social contacts during the pandemic due to fear of infection^38^, we think that health professionals interacting with cancer patients and survivors during and beyond the pandemic should consider assessing suicidality.

Within the regression models that also included sociodemographic characteristics, cancer was an independent risk factor for anxiety symptoms within the whole sample. Furthermore, the study gave insight into risk and protective factors of other domains of life that interacted with cancer. We found that a lower household income increased cancer survivors’ risk of anxiety symptoms. There is previous evidence from an early phase of the pandemic that financial difficulties contributed to US-American gynecologic cancer patients’ worries and anxiety^65^. In a sample of 394 Australian patients living with hematological cancers, financial concerns (including income losses) were associated with higher levels of psychological distress (operationalized as the Kessler 10-item assessment)^66^.

We also observed moderating effects of gender. While female gender was associated with higher levels of anxiety within the overall sample, in contrast to our hypothesis, there was an interaction of cancer and gender in the sense that men with cancer were particularly at risk for experiencing higher levels of anxiety. Comparable results were previously reported based on a large, German community cohort^22^. These findings illustrate that the investigation of gender-dependent associations, e.g., of chronic illness and well-being, is informative (compared to modeling gender as an adjustment variable/solely controlling for it as a potential confounder). As women generally report higher levels of mental distress than men, particularly internalizing symptoms including depression and anxiety symptoms^67^, such specific risk constellations for men might otherwise not be detected. Possible explanations for the observed stronger relation of cancer and distress in men could be underlying sociocultural differences between men and women that shape a person’s perception, emotions and behavior, such as internalized gender norms: For men, traditionally masculine gender role socialization, respectively hegemonic masculinity emphasizing toughness, physical strength and self-reliance may impede acknowledging and accepting distress^68^. Previous research has found that male cancer patients are less likely to utilize the psychosocial support offers which are available in the context of German standard care than female cancer patients, even if they are suffering emotionally^69^. In addition, public health reporting during the COVID-19 pandemic has characterized men as a risk group for worse outcomes if infected with SARS-CoV-2^70,71^, citing both biological and behavior/lifestyle differences between men and women. It is possible that men living with cancer were particularly worried by such news. Along these lines, an Israeli population study underscored the role of perceived susceptibility in shaping emotional reactions to the pandemic threat^72^.

While the present study cannot give insight whether respective cognitions were relevant for cancer survivors during the pandemic, it showed that biological, social and psychological variables were all involved in participants’ mental health outcomes. In particular, loneliness emerged as an important, potentially modifiable risk factor. Hence, as the pandemic continues and restrictions such as physical distancing measures remain in place, fostering social connectedness – especially in at-risk groups such as cancer survivors – should be a priority of public health interventions. These could, for instance, include “support bubble” solutions (as recommended by the UK Government^73^) or harness recent advances in digital communication and e-health applications.

### Strengths and limitations

The use of a representative, population-based survey has strengths compared to study designs that invite an oversampling of cancer patients and survivors who are distressed (for instance through counseling centers, where one would primarily meet individuals in need of support) and to the use of convenience samples assessed online (which cannot capture the situation of individuals without internet access and with low digital literacy, potentially leading to the systematic exclusion of older and/or especially vulnerable populations in the context of the pandemic^74^). However, there are also drawbacks to the present approach, such as the relatively small proportions of distressed individuals and the small number of cancer survivors. Future research efforts should survey larger samples of individuals living with cancer. They should also capture potential cancer-specific (such as time since diagnosis, cancer type and stage) and pandemic-specific risk factors (such as experienced treatment delays/disruptions, difficulties obtaining health care, perceived risk of infection, concerns about loved ones, and the like). The lack of such information and the unavailability of a comparison sample assessed before the pandemic (using a comparable methodological approach) present limitations of this study.

Beyond that, given the scarcity of intersectional, in particular gender-sensitive/-specific research in the context of the COVID-19 pandemic e.g.,^75^, we think that the empirical investigation of interaction effects is a strength of the present study. Like the rest of society, individuals with cancer may be vulnerable to psychological distress or be protected from it, respectively, because of their personal characteristics and their life circumstances. Furthermore, the latter may also be causally related to the disease (see e.g., the financial burden of cancer treatment and care which is often summarized as “financial toxicity”^76^). Therefore, including information beyond directly disease-related aspects is useful in order to identify particularly vulnerable subgroups. Respective findings can inform targeted prevention and intervention efforts in the community and/or medical settings.

We used only one item (drawn from the PHQ-9) to measure suicidal ideation and screening instruments (PHQ-2 and GAD-2) to assess depression and anxiety symptoms. Given the study design, it was not feasible to include longer questionnaires or clinical interviews although these methods are superior in identifying individuals at risk e.g.,^77^. With respect to the cancer diagnoses, there was no information about the disease entity/stage/treatment phase. Larger studies that include these aspects are able to ascertain the risk for mental distress by cancer type e.g.,^37^; this would be especially useful to guide screening and prevention efforts in clinical practice. Lastly, the present study captured only the first wave of the COVID-19 pandemic in Germany. Both levels of distress and specific risk factors for distress might change as the pandemic continues.

## Conclusions

Using data from a representative population survey conducted during the COVID-19 pandemic, the present study showed associations of cancer and psychological distress, in particular anxiety symptoms. The findings also indicated that the importance of cancer for a person’s mental health is modified by individual differences and sociodemographic characteristics, such as a person’s gender and financial situation. Within the context of the pandemic, especially economically disadvantaged and male cancer survivors could be in need of more social and/or professional support. Public health measures aimed at containing the spread of the virus should be adapted accordingly to safeguard vulnerable individuals’ mental health.

## Data Availability

Data is available from the corresponding author upon reasonable request.
